# Good Thinking or Gut Feeling? Cognitive Reflection and Intuition in Traders, Bankers and Financial Non-Experts

**DOI:** 10.1371/journal.pone.0123202

**Published:** 2015-04-13

**Authors:** Volker Thoma, Elliott White, Asha Panigrahi, Vanessa Strowger, Irina Anderson

**Affiliations:** 1 University of East London, London, United Kingdom; 2 Canterbury Christ Church University, Canterbury, United Kingdom; 3 University of Glasgow, Glasgow, United Kingdom; 4 Eclipse Energy Group, London, United Kingdom; University Hospital of Bellvitge-IDIBELL; CIBER Fisiopatología Obesidad y Nutrición (CIBERObn), Instituto Salud Carlos III; Department of Clinical Sciences, School of Medicine, University of Barcelona, Spain, SPAIN

## Abstract

The current study investigated differences in decision-making style and risk-taking between financial traders, non-trading bank employees, and people not working in finance. Traders scored significantly higher than participants in the other two groups on the cognitive reflection test (CRT) which measures the tendency to inhibit automatic but frequently false responses in reasoning tasks. Scores for traders compared to people outside the banking sector were also higher on a self-rated scale for reflective thinking in decision-making, but there were no differences in self-rated intuitive thinking between groups. Financial risk-taking correlated with cognitive reflection scores and was significantly lower in the non-expert group compared to the other groups working in financial services. Traders in the current study showed no elevated preference to use ‘intuition’ in their decision-making compared to other groups. Overall, these results indicate that compared to non-expert participants financial traders have a higher self-rated tendency for reflective thinking and a greater propensity to inhibit the use of mental shortcuts (heuristics) in decision-making.

## Introduction

Are financial experts good decision-makers? This question has become a major focus of public and scientific attention in the decade following global market phenomena such as the “dotcom” bubble and the 2007 financial credit crisis [1]. Good decision-making is traditionally equated with analytic rationality [2] and contrasted in much of experimental psychology with the use of ‘heuristics’—simple decision rules that produce systematic biases away from normative decision outcomes [3], [4] (see [5] for an argument that heuristics may be the result of adaptations to natural and social environments). Heuristics are often described as mental ‘shortcuts’ or rules of thumb that reduce the complexity of judging and deciding between certain alternatives. For example, human judgment regarding the likelihood of certain events is readily influenced by how easily previous examples of that event are retrieved from memory [6], and the willingness to pay for goods can be influenced by arbitrary values of unrelated variables [7]. Therefore, tasks typical for financial experts such as judging risks and pricing of investments may be susceptible to heuristics as well. Here, we ask whether judgment and decision biases are as common in traders and bankers as they appear to be in the general population, and whether these are related to differences in cognitive ability (‘good thinking’) or rather differences in thinking and decision making styles (such as relying on one’s ‘gut feeling’).

Over the last four decades a number of heuristics and resulting biases in judgment and decision making have been demonstrated in financial traders (for overviews see e.g., [1], [8]). For example, Barber and Odean [9] and Chen and colleagues [10] found that traders are susceptible to the disposition effect, overconfidence, and the representativeness bias. Consequently, many contemporary scholars in behavioural finance [11]–[13] espouse the view that people working in financial markets are frequently not thinking or acting rationally or ‘analytically’, at least in part because they are susceptible to decision biases based on heuristic thinking [8]. At the same time, however, there is general evidence that expertise seems to guard against biases in judgments [14]. In the financial domain, Shapira and Venezia [15] report that professional investors are less affected by the disposition effect—the tendency to hold on longer to falling stocks than winning ones—compared to individual investors [16]. Thus, the research literature does not give a clear picture to what degree financial experts are closer to normative judgments (i.e. less biased) than lay people.

To address the question of whether traders’ expertise in decision-making is due to higher reflective capabilities (and a reluctance to rely on heuristics) or not, we use methods from a popular framework in the judgment and decision-making literature which contrasts analytic deliberation (‘good thinking’) with intuitive (‘gut feeling’) inferences [17], [18]. This growing research area (also known as the dual systems framework [3], [19]) provides evidence that human reasoning and decision-making are the product of two cognitive systems (Recently, the term “System” has been criticised as it implies different “hardware”, thus the more neutral terms “Type 1” and “Type 2 processing” are sometimes used [20] to include the notion of different process (software) that can operate under different systems. We retain the term “System” here for simplicity.). System 1 is described as a fast, effort-free collection of autonomous processes that give rise to ‘intuitive’ judgments resulting in decision biases, i.e. the outcomes are substantially suboptimal or simply wrong compared to normative standards [17], [21]. System 2, in contrast, is characterised by slow, effortful, deliberative, and rule-based reasoning that conforms more closely to normative notions of rationality. In contrast to System 1 and its use of heuristics, the deliberative system of reasoning (System 2) is associated with reflective, primarily verbal, and mostly conscious processing [17], [22]. In addition, System 2 is thought to be able to override or intervene on automatic (System 1) responses and thereby guards against biases in decision making resulting from people’s use of heuristics [4], [23].

To investigate what happens when System 1 thinking (i.e., using heuristics) is in conflict with more reflective thinking, Frederick [23] developed the Cognitive Reflection Test (CRT). High performance in this test is associated with an ability to override intuitive System 1 responses and engage in analytic thinking, which leads to reduced biases and more normative responses. The CRT has been found to uniquely predict performance on heuristics-and-biases tasks after other measures of individual differences (including IQ) had been taken into account [24]. The CRT score for individuals is also associated with extended delay of gratification and normative responses to risky choice options in gambles [23]. Therefore, our first prediction is that if financial expertise is based on ‘good thinking’ then financial experts such as traders should score higher on the CRT than non-experts, indicating reduced reliance on heuristics.

Although cognitive ability is an important predictor of reflective thinking, the CRT correlates only moderately with other measures of cognitive ability [23]. In a comprehensive study, Toplak and colleagues [24] identified additional factors alongside an individual’s algorithmic capacity- including executive functions, thinking dispositions, as well as a unique contribution from the CRT itself. To tap into ‘good thinking’ that is further dissociated from cognitive ability alone, we included therefore an attitudinal concept of rational thought—cognitive style. The term cognitive style [25] refers to one’s proclivities in information processing based on how one perceives, conceptualises, and stores information. A well-studied framework for cognitive style is the cognitive-experiential self-theory by Epstein (CEST; [26]). Epstein conceptualises thinking styles as either “experiential” or “rational processes”, that is whether one habitually responds *primarily* intuitively or *primarily* reflectively in decision-making situations. Based on CEST, Epstein and colleagues have developed a questionnaire to measure a person’s cognitive style: the Rational Experiential Inventory (REI; [27]). The REI has a relatively high degree of reliability and validity for the underlying constructs [28], [29] with rational (REI-R) but not experiential thinking (REI-E) being positively correlated with normative responses in judgment tasks (including the CRT). Thus, our second hypothesis is that if financial experts rely on ‘good thinking’ then they will score higher than non-experts on the REI scale for rational thinking (REI-R).

Unlike reflective/analytical thinking, intuition is often generally conceived of as mental processes leading to decisions that are made ‘‘with no awareness of the rules or knowledge used for inference and can feel right despite one’s inability to articulate a reason” ([30], p. 64). One explanation of how ‘intuition’ may be explained is based on a substantial body of work suggesting that fast and simple heuristics can lead to surprisingly accurate inferences under certain conditions [31], [32]. In the financial domain, evidence suggests decision-makers are using ‘intuition’ or ‘gut feeling’ successfully. For example, Betsch and colleagues [33] report that their participants engaged in ‘unconscious’ or implicit valuation of stock share values that had been presented to them earlier. Recently, surveys [34] and interview studies [35], [36] have also reported widespread use of ‘intuition’ in expert financial decision-making, at least in the sense that assessment of financial data was integrated with a reliance on gut feelings. Thus—in contrast to predictions from the dual systems framework—‘gut feelings’ and ‘intuition’ can be considered potential useful mechanisms or cognitive adaptations to the tasks traders have to regularly perform [36], [37]. For this reason, we have included two different expert groups (traders and non-trader bankers) to be compared with a sample of people not related to the financial world. If traders used intuition (i.e. fast, effort-reducing heuristics [38], [39]) we would expect them to score higher on the REI-E than the non-trading bankers as well as a non-financial sample (see Fenton O’Creevy et al. [37] for an in-depth account of the trading profession and its environmental context).

The final variable of interest was risk-taking in different domains (e.g economic, health, social etc.). Risk-taking is mediated by a multitude of factors, such as risk perception (the extent to which one is aware of a risk), risk attitude, risk propensity, emotion, personality, and many others (for reviews see [1], [40]) which renders an adequate account of the literature outside the scope of the current article. Our main interest was in the difference between financial risk-taking scores in each participant group and its relationship with the CRT and cognitive style. In line with the dual systems framework, we predicted that financial risk-taking should be correlated with CRT scores (see also Frederick [23] who found less risk aversion in high CRT scorers when proposed with high and medium stakes gambles).

To our knowledge, one striking omission in previous research on financial experts is that the seminal work on CRT and REI has not been examined in relation to professional financial traders in particular (including risk-taking). We therefore also measured some commonly used trader performance indicators (financial risk-taking, salary/bonus, number of transactions).

We conducted this investigation in two parts: Study 1 comprised a detailed questionnaire for financial traders, which included the variables described above, including those related to trading. In Study 2, we used a shorter questionnaire concentrating on the main variables of thinking style, cognitive reflection, and risk-taking for two other participant groups. One group consisted of general banking workers, the other comprised members of the general public working outside finance. This allowed us to compare all three groups—traders, bankers, and non-experts—in a final step to establish whether they differ in the use of reflective thinking or intuition. In brief, we found that while traders were no different in their self-reported use of ‘intuition’ or ‘gut feeling’ to other people, they showed higher propensity and ability to engage in reflective thinking than the participants without a finance background, and also scored higher than non-trading bankers on self-rated rationality in their thinking style.

## Study Structure and Statistical Analysis

We first present the survey run on a sample of professional traders (Study 1) and focus on presenting the Pearson’s correlations between the main variables of cognitive thinking as well as important demographics (including length of experience, income, and levels of education). We then present the results of Study 2 with a similar analysis of correlations in the two non-trader samples (banking staff and the general population). In a final step, we compared the three groups by running a MANOVA on the main dependent variables of interest (reflective thinking, thinking styles, and risk-taking). To follow-up significant differences between group means ANOVAs were used, and pairwise comparisons were corrected by using the Games-Howell procedure (as appropriate for unequal sample sizes).

## Study 1—Method

### Participants

A total of 44 participants (M = 34.3 years, SD = 7.6), from trading desks of four UK investment banks and trading houses (in London), were recruited for Study 1. The organisations were approached using e-mail via their official contact details, and in one case talking to a desk manager at a decision-making conference, to grant us permission to contact their traders directly (via e-mailed letters explaining the broad aims of the study). The group was predominantly male (88%) and highly educated (over 75% with a first or post-graduate degree). Participants were recruited directly through emails, or in coordination with trading desk managers of financial organisations. All participants completed an online questionnaire, which comprised several inventories and tasks, detailed below.

### Ethics Statement

The study was approved by the Ethics committee of the University of East London and participants were informed at the beginning of the questionnaire about their right to withdraw from the study at any time. Consent was given written (electronically using the online questionnaire, ticking a box which indicated they had read the information and consented to take part).

### Measures and procedures

The online survey was hosted on a commercial survey platform (www.surverygizmo.com). The questionnaire comprised various webpages on which participants were asked to respond to the following sections: General information (including ethical approval and consent form), demographics, qualification (scored from 0 to 5, with no qualification as the lowest score, PhD as the highest), extended career information (including years of experience, salary), the Cognitive Reflection Task [23], the Rational-Experiential Inventory [27], and a measure of domain specific risk-taking [41]. We also assessed two personality variables but these were not part of the current hypotheses and are not further reported.

The CRT [23] comprises three questions in which people have to make a judgment, designed to test the propensity to engage in heuristic thinking, or rather, the failure to intervene in a rapid, default, heuristic processes. One example is: “A bat and a ball cost $1.10. The bat costs $1.00 more than the ball. How much (in cents) does the ball cost?”. According to Frederick [23] an intuitive answer to questions like these springs to mind (for most people, it is the answer “10 cents”), whereas if people would engage in more analytic (deliberate) reflection, the correct answer should emerge as “5 cents”. Questions are scored as either correct or incorrect and summed across the three items. Liberali and colleagues [42] report a moderate to high (Cronbach’s alphas between 0.64 and. 74) reliability. The CRT was modified in this study by adding a fourth question: “Have you been asked any of these questions before?” A positive response to this question was a cue for a participant’s CRT scores to be excluded, to guard against possible knowledge effects.

The Rational-Experiential Inventory is available in 3 versions: REI-40 and its shorter form, the REI-24 [27], and a 10-item version [43]. The REI-24 was used here to measure thinking/cognitive style. The instrument comprises items from the original Need for Cognition Scale (NFC [44]) which relates to deliberative (System 2) thought (also called REI-R, for rationality), and several Faith In Intuition (FI) items [43] which assesses intuitive-experiential tendencies (or REI-E, for experientiality). An example of a REI-I question is: “Thinking hard and for a long time about something gives me little satisfaction”. To summarise, the REI-24 consists of two unipolar scales (12 items each) which put participants on two dimensions of decision making style. The first scale measures engagement in and favourability of cognitive activities and corresponds to deliberative thinking, whereas the second scale measures favourability and self-perceived engagement (or ability) of intuitive thinking. Previous studies for the original [27] and the 24 item version [45] report a high internal consistency within the REI scales (Cronbach’s alphas >0.85).

The domain-specific risk-attitude scale (DOSPERT [46]) assesses risk-taking and perception across six domains: Career, Recreational, Health, Safety, Social and Financial. In Study 1, only the financial domain of the DOSPERT (risk-taking) was used in full with 6 specific items, relating to three on gambling and three on investment decisions. An example of an item is: “How likely and how risky is investing 5% of your annual income in a very speculative stock?”. These six specific items were used alongside five items covering the remaining five domains a total of eleven items. The latter five general or ‘domain-level’ risk items were taken from the Risk-taking Index developed by Nicholson and colleagues [47]. Participants provided a likelihood assessment (using a 7-step Likert scale) for each item that they would engage in the risky behaviour. Scores were averaged for an indication of overall financial and non-financial risk-taking.

## Study 1—Results

Raw data were screened for missing values and possible outlier influences. Seven traders indicated in the questionnaire to have previously seen at least one of the CRT questions and these data points were removed from analysis. There were 24 traders who opted not to answer the question on earnings. Finally, one response to the question about number of transactions (M = 83.19, SD = 216.62) was removed as an outlier (50,000 transactions were indicated in this case, which is either suggestive of an error or relates to computer-based trading). Responses from the online questionnaire were first analysed for intercorrelations between variables, shown in Table 1.

**Table 1:** Two-tailed correlations between main measures in the trader group (Study 1, n = 44).

	1	2	3	4	5	6	7	8
1. Years trading	-							
2. Qualification	.55**(44)	-						
3. Total salary	.56**(43)	.02 (20)	-					
4. Transactions/day	.25 (43)	-.25 (43)	.21 (20)	-				
5. Financial Risk	-.12 (43)	.04 (20)	-.10 (19)	.26 (42)	-			
6. Non-financial risk	.06 (43)	-.12 (19)	.29 (19)	.12 (42)	.37*(43)	-		
7. CRT	.20 (37)	-.09 (17)	.37 (17)	.15 (36)	.05 (36)	.31 (37)	-	
8. REI-R	-.34*(44)	.35*(20)	.12 (20)	-.06 (43)	.31*(43)	-.24 (43)	-.04 (37)	-
9. REI-E	.37*(44)	-.23 (20)	.34 (20)	-.20 (43)	-.16 (43)	.20 (43)	.07 (37)	-.31*(44)

Notes. REI-R = Rational-Experiential Inventory—Rational subscale score; REI-E = Rational-Experiential Inventory—Experiential subscale score; CRT = Cognitive Reflection Test; Scores for 5–11 were on a Likert-scale between 1 (minimum) to 5 (maximum). CRT scores were between 0–3.

** = p<.01;

* = p<.05.

Age correlated highly with years of trading experience, *r(*42) = .991; *p*<.001 and its correlations with other variables (all equivalent to experience in terms of significant correlations) are therefore not reported separately in Table 1. Experience (number of years trading, M = 10.16, SD = 7.96) correlated negatively with qualification (highest educational degree achieved, M = 3.18, SD = 0.81), *r(42)* = -.55, *p*<.001, which may suggest a trend over recent years towards hiring more post-graduates as traders than in previous years ([37]; see General Discussion). Salary data (including bonus) were only reported from less than half the traders (M = 393,865.00, SD = 387,902.22), and they correlated positively with years of experience only, *r(18)* = .56, *p* = .01.

General (non-financial) risk-taking (M = 2.95, SD = .79) correlated only with financial risk-taking (M = 3.89, SD = 1.41), *r(43)* = .37, *p* = .01. Scores for the REI-R (M = 3.97, SD = .55) correlated with financial risk-taking, *r(43)* = .31, *p* = .04, qualifications, *r(43)* = .35, *p* = .02, and correlated negatively with years of experience, *r(43)* = -.34, *p* = .02. REI-E (M = 3.63, SD = .58) scores correlated positively with experience, *r(43)* = .37, *p* = .01. In addition, scores for the REI-E correlated negatively with REI-R, *r(43)* = -.31, *p* = .04. There were no significant correlations involving number of transactions per day (M = 1217.66, SD = 7528.28). Rationality, as measured by REI-R showed a Cronbach alpha of. 88, while the Experientiality (REI-E) subscale returning a reliability coefficient of. 90. The six specific items on financial risk-taking from the DOSPERT revealed sufficient reliability (Cronbach’s alpha = .79). The five items covering the remaining five domains had a Cronbach’s alpha of. 50. For the CRT, there were no significant correlations, which may be due to a ceiling effect as most traders scored highly or very highly (M = 2.73, SD = 0.61, of a maximum score of 3). There was, however, a (non-significant) numerically moderate correlation with salary (*r* = .37, *p* = .15), which was based on only 17 participants.

In general, there is clear evidence of cognitive style relating to a number of variables in our sample of traders. Traders who preferred a ‘rational’ style tend to have less years of experience but a higher level of qualification. This may be an adaptation to task demands or a cohort effect and related to the way traders are recruited, a point which we will revisit in the General Discussion. Importantly, we found that cognitive style (REI-R) did correlate with a variable which is important for traders’ tasks: financial risk-taking. Finally, there was a numerical trend for a correlation between CRT score and salary. Study 2 investigated cognitive style and cognitive reflection across roles (and therefore less specialised than traders) in the financial sector as well as a control group comprising employees in a range of non-banking jobs. This finally allowed us to make comparisons between all groups.

## Study 2—Method

### Participants

Participants were recruited using three methods, each of which linked to a web-survey hosting site (see Measures and Procedure). Contacts known to the third author, who worked in banking related jobs, were emailed with study information to allow informed consent. They were also asked to share the invitation with colleagues. To find members of the non-banking group online psychology research forums and social networks were used to place advertisements. Recruitment using research forums and social networks complied with relevant ‘Terms of Service’. A total of 110 participants took part in Study 2, with 53 (14 of whom were females) participants coming from a range of jobs in banking and financial services (14 identified themselves as financial traders in the questionnaire although we were not able to verify their roles) and another 57 participants from jobs not related to finance were recruited (using an opportunity sample based on e-mails and facebook messages). There were 45 females in the overall sample. Ages ranged from 18 to 71 (*M* = 33.4, SD = 10.56) and the sample was well educated, compared to the general population (89% had at least a first university degree). There were no significant age differences between banking (M = 33.0) and non-banking (M = 33.7) groups.

### Measures and procedures

The online survey was hosted on a commercial survey platform (www.surverymonkey.com). As in Study 1 the relevant ethics and consent considerations were applied here. As in Study 1, the questionnaire comprised several webpages on which participants were asked to respond to the following sections: General information, the CRT, the REI, a risk-taking questionnaire, and questions regarding basic demographic and employment information. Note that data collection for Study 2 was actually begun before Study 1 had commenced. Because of concerns for potential low return rates we used shortened or adapted versions of the scales described in Study 1.

The REI-10, the short form of the Rational-Experiential Inventory [43] was used here. Like the longer form of the REI used in Study 1, the REI-10 comprises an equal number of items for rational and experiential thinking styles [43]. The internal-consistency reliability coefficient of the REI-R has been previously reported as 0.73, and 0.72 for the REI-E. These values are usually deemed consistent with longer forms [43].

Risk-taking was measured using a self-report measure using 12 items, in six domains of risk-taking: financial (see Study 1), health, safety, recreational, career and social on a 5-item Likert scale. Two questions were asked of each domain, utilising those used in the original DOSPERT by Weber, Blais, and Betz [41]. One question on financial risk-taking was modified to a composite of the 3 questions on financial gambling and betting: “Betting a day's income in a poker game, casino or sporting event.” (equivalent to the separate items in that category used in Study 1).

## Study 2—Results

Table 2 shows the summary of scale means and standard deviations in Study 2 for the two groups of non-banking and finance-related participants.

**Table 2:** Mean scores (and standard deviations) for risk-taking, cognitive reflection, and propensity for rational vs. experiential thinking style in Study2.

	Overall (N = 110)	Male (n = 55)	Female (n = 45)	Finance workers (n = 53)	Non-banking (n = 57)	p-value (group differences)
CRT score	1.47 (1.1)	1.58 (1.1)	1.31 (1.04)	1.62 (1.1)	1.33 (1.06)	.20
REI-R	3.72 (0.65)	3.8 (0.62)	3.61 (0.67)	3.76 (0.60)	3.67 (0.70)	.65
REI-E	3.64 (0.64)	3.7 (0.59)	3.6 (0.72)	3.74 (0.58)	3.55 (0.69)	.13
Non-financial risk-taking	3.19 (0.56)	3.28 (0.59)	3.07 (0.49)	3.37 (0.49)	3.03 (0.57)	.02
Financial risk-taking	2.69 (0.98)	2.93 (0.96)	2.34 (0.91)	3.06 (0.86)	2.36 (0.95)	.003

Notes. Standard deviations are in parentheses. REI-R = Rational-Experiential Inventory—Rational subscale score; REI-E = Rational-Experiential Inventory—Experiential subscale score; CRT = Cognitive Reflection Test. Scores range form 1 (minimum) to 5 (maximum) except for CRT (0–3) for male (n = 55) and female (45) participants employed in banking (n = 53) or outside the finance sector (n = 57).

A correlational analysis explored potential relationships between the variables described in the methods, including age. REI-R scores correlated with CRT performance scores, *r(*108) = 0.33, *p*<0.001, and with non-financial risk-taking, *r(*108) = .20, *p* = .04, but not with financial risk-taking, *r(*108) = .12, *p* = .23. CRT correlated positively with financial risk-taking, *r(*108) = .21, *p* = .03, and non-financial risk-taking, *r(*108) = .22, *p* = .02. Participant’s age only correlated significantly—negatively—with non-financial risk-taking, *r(*108) = -.34, *p*<.001, indicating that with increasing age risk-taking decreases in our sample. The only other significant (or even marginally significant) correlation was between the two risk-taking scores, *r(*108) = .37, *p*<.001. Thus, these results confirm earlier findings that CRT correlates with REI-R ([42] and risk-taking measures (e.g., choosing a gamble with a higher expected value than a sure win [23]).

To explore the effect of the factor participant group on the five dependent variables of interest (CRT, REI-R, REI-E, non-financial and financial risk-taking) a two-way MANOVA was performed with the variables group (of participants) and sex as fixed factors (sex has previously been shown to be a factor in CRT scores and risk aversion [23]), and age and education as covariates (Box’s assumption of equality of covariance matrices was supported, *p* = .794). There was a main effect for the factor participant group, Wilks’ λ = .883, *F(*5, 100) = 2.66, *p* = .27, partial eta2 = .12, but not for sex, Wilks’ λ = .918, *F(*5, 100) = 1.80, *p* = .12, partial eta^2^ = .08, nor the interaction of group and sex, Wilks’ λ = .980, *F(*5, 100)<1. Of the covariates only age had a significant effect, Wilks’ λ = .841, *F(*5, 100) = 3.78, *p* = .004, partial eta^2^ = .16,

Follow-up ANOVAs for the factor participant group revealed only a significant effect for the dependent variables non-financial and financial risk-taking, *F*(1,104) = 5.63, *p* = .02, partial eta^2^ = .05, and *F*(1,104) = 9.46, *p* = .003, partial eta^2^ = .08. Participants from the banking sector scored higher on both types of risk-taking than the non-banking group. There were no significant differences between groups in cognitive reflection as measured by CRT performance, *F*(1, 104) = 1.61, *p* = 0.20, nor were there any differences in the REI-R scores, *F*(1, 104)<1, or REI-E scores, *F*(1, 104) = 2.37, *p =* .*126)*. This indicates that there were no differences in cognitive style as measured by the REI between the two groups.

A significant difference between groups was observed with respect to income. Self-reported income was measured via categorical steps of £20,000 increments. Annual income differed between groups, with 72% of financial sector workers’ earnings in the highest income bracket (£60,000 and above), while only 12.1% of the non-banking sample's earnings reached this category. This was a significant difference between groups, *X* ^2^ (1, 108) = 45.58, *p*<0.01.

The result of Study 2 showed that the only significant differences between the two groups of bankers and non-experts (other than income) was that of risk-taking, with participants employed in the financial sector scoring higher in risk-taking than the non-banking group. Although males were previously found to be more risk seeking than females [48] we did not find reliable sex differences here. While the groups did not differ in their CRT scores or cognitive style, cognitive reflection and risk-taking were positively correlated, as were CRT scores and the need for cognitive thinking.

## Comparison of All Groups

The final analysis contrasted participants’ data from Study 1 and 2, using data collected from all participants and divided into three groups—‘traders’ (Group 1), non-trading professionals working in ‘banking’ (Group 2), and people with no financial work background (Group 3). Participants in Group 3 (n = 57) were respondents who do not work in banking or the financial industry, as described in Study 2 (‘non-banking’). Participants in Group 2 (n = 39) were those who work in a range of financial roles in banks and related organisations (i.e. the majority of the banking group participants in Study 2), but not in trading or trading support roles. Participants in Group 1 (n = 58) were financial traders, which comprised those from Study 1 (n = 44) and 14 cases from the financial worker group in Study 2 (who had identified themselves as financial traders in the questionnaire). As a consequence, the latter sub-group had not received the same extended version of the questionnaire as the traders in Study 1. The current analysis is therefore limited to the four main variables that were measured with equivalent scales in all groups: Rationality (REI-R), experientiality (REI-E), financial risk-taking, and CRT (the rating scales for financial risk items used in study 1 and 2 differed in length—5 point vs. 7 point—and therefore a transformation was used [49]).

Table 3 shows the correlations between the main measures across all three groups. There were only four reliable correlations: experientiality (REI-E) correlated positively with age, *r*(152) = .16, *p* = .050), REI-R correlated positively with financial risk-taking, *r*(151) = .18, *p* = .03. CRT scores correlated positively with rationality (REI-R), *r*(145) = .30, *p*<.001, and financial risk-taking, *r*(144) = .19, *p* = .02.

**Table 3:** Intercorrelations for main measures across all participants groups (n = 152) in Studies 1 and 2.

	Age	Qualific.	Fin. Risk	REI-R	REI-E	CRT
Age	-					
Qualification	-.02	-				
Fin. Risk-taking	-.03	.05	-			
REI-R	-.09	.12	.18*	-		
REI-E	.16*	.00	-.01	-.08	-	
CRT	-.02	-.00	.19*	.30**	-.03	-

Notes: Standard errors are in parentheses. REI-R = Rational-Experiential Inventory—Rational subscale score; REI-E = Rational-Experiential Inventory—Experiential subscale score; CRT = Cognitive Reflection Test; Scores for 5–11 were on a Likert-scale between 1 (minimum) to 5 (maximum). CRT scores were between 0–3.

** = p<.01;

* = p<.05.

To investigate the effect of participant group on the four dependent variables of interest (CRT, REI-R, REI-E, and financial risk-taking) a 2-way MANOVA was performed with the independent variables participant group and sex (previous studies suggest this as a variable, e.g.,[23]) as factors, and age and education as covariates. Box’s assumption of equality of covariances was upheld, *p* = .661. There was a main effect for the factor participant group, Wilks’ λ = .85, *F(*8, 270) = 2.94, *p* = .004, partial eta^2^ = .08, but not for sex, Wilks’ λ = .961, *F(*4, 135) = 1.37, *p* = .25, partial eta^2^ = .04, nor the interaction of group and sex, Wilks’ λ = .97, *F(*8, 270)<1, or any of the covariates, *F*s <1.54.

Follow-up between-groups ANOVAs were run to investigate group differences for the following variables—financial risk-taking, REI-R, REI-E, and CRT. Table 4 shows the means and standard deviations for the four scales of interest. A between-group ANOVA with CRT scores as dependent variable revealed a significant effect of group, *F*(2, 154) = 19.53, *p*<.001, partial eta^2^ = .21. Post-hoc pairwise comparisons using the Games-Howell procedure revealed that traders scored higher compared to both banking (*p*<.001) and non-banking (*p*<.001) participants. There was no difference between the latter two participant groups, *p* = .98.

**Table 4:** Means and standard deviations for risk-taking, cognitive style, and cognitive reflections scores across all three groups.

	Group 1 Traders (n = 58)	Group 2 Banking (n = 39)	Group 3 Nonbanking (n = 57)	p-value (Group 1 vs. 2)	p-value (Group 2 vs. 3)
REI-R	3.95 (0.57)	3.71 (0.58)	3.67 (0.69)	.11	.95
REI-E	3.66 (0.59)	3.73 (0.58)	3.55 (0.69)	.84	.34
CRT score	2.49 (0.88)	1.54 (1.07)	1.33 (1.06)	.000	.62
Financial Risk-taking	3.05 (0.97)	2.91 (0.82)	2.35 (0.95)	.71	.008

Notes: Standard errors are in parentheses. REI-R = Rational-Experiential Inventory—Rational subscale score; REI-E = Rational-Experiential Inventory—Experiential subscale score; CRT = Cognitive Reflection Test; Scores for 5–11 were on a Likert-scale between 1 (minimum) to 5 (maximum). CRT was scored were between 0–3. P-values for group differences are given for pairwise comparisons (Games-Howell corrected).

The ANOVA with REI-R as dependent variable found also a significant effect of group, *F*(2, 154) = 3.40, *p* = .036, partial eta^2^ = .04. Pairwise comparisons showed that traders scored higher compared to non-banking (*p* = .047) but not banking (*p* = .106) participants. There was no difference between banking and non-banking participants, *p* = .95. The ANOVA on REI-E scores revealed no difference between groups, *F*(2, 154)<1.1.

Finally, an ANOVA on financial risk-taking scores revealed a significant effect of group, *F*(2, 154) = 8.912, *p*<.001, partial eta^2^ = .11. Again, traders scored significantly higher compared to non-banking (*p*<.001) but not banking (*p* = .72) participants, and banking participants scored higher than non-banking, *p* = .008. Because items from different versions of the DOSPERT were used in Study 1 and 2, we ran an additional analysis, only including a subset of questions on financial betting or gambling (e.g., “Betting a day’s income on the outcome of a sporting event”) that overlapped in both studies. We re-ran the MANOVA as above but including the mean score for the financial betting items in lieu of the financial risk-taking composite. The results were comparable to the findings above, with a main effect for the factor participant group, Wilks’ λ = .85, *F(*8, 270) = 2.75, *p* = .007, partial eta^2^ = .07, but not for sex, Wilks’ λ = .961, *F(*4, 135) = 1.40, *p* = .24, partial eta^2^ = .04, nor the interaction of group and sex, Wilks’ λ = .97, *F(*8, 270)<1, or any of the covariates (there was a trend for age, Wilks’ λ = .939, *F(*4, 135) = 2.19, *p* = .074). Follow-up tests confirmed the pattern of results found with the complete set of items on financial risk-taking.

In summary, traders favoured a more reflective approach, scored higher on the CRT, and were more financially risk-taking than the non-banking participants. ‘Banking’ workers (who were not traders) scored higher only in financial risk-taking than non-bankers, while they trailed traders significantly in performance on the CRT. These results seem not to be confounded by differences in sex, age, or education. However, there is a general indication in the literature of an effect of sex as well as cognitive ability on CRT scores [23]. To further clarify whether a combination of these effects may account for the differences in the CRT scores, we ran an additional non-parametric analysis and included only cases with high scores (with participants having answered 2 or all 3 of the CRT questions correctly, as was the case for the great majority of traders). A Chi-square analysis on these high-scoring participants revealed an association between group and category of response (2 vs. 3 items correctly answered), *X* ^2^(2, 1) = 9.49, *p*<0.01, with 75% of the traders having answered all items correctly, whereas only 50% of the remaining banking and 40% of non-banking male staff achieved the highest score (see Table 5). Thus, even when the CRT results were controlled for sex differences and the analysis limited to high-scoring male participants, traders’ scores were still significantly superior to the males in the other groups.

**Table 5:** Crosstabulation of cognitive reflection scores (CRT) counts for high-scoring males between all groups.

		Industry			
		Trader	Banking	Non-Banking	Total
CRT Score	2 items correct	8	7	9	24
	3 items correct	32	7	6	45
	Total correct	40	14	15	69

## Discussion

The current study asked whether there are differences in analytic thinking ability (cognitive reflection), decision-making style (rational versus experiential), and financial risk-taking between financial traders, a non-trader sample employed in the banking sector, and a general population group. The hypotheses predicted that if good thinking underlies financial traders’ expertise this should be dependent on presumed System 2 operations—either in the form of higher cognitive reflection ability, including a higher ability to monitor and interrupt heuristic processes [23], [50], or by adopting a cognitive style that preferentially engages System 2 [27], [43]. As predicted by our first hypothesis, traders scored significantly higher on the CRT than the other participants in the banking or non-banking sector. Based on the literature on the CRT [23], [24] this indicates that traders are generally less susceptible to using mental shortcuts (heuristics) in solving judgment problems of this type, and they are relying more on System 2 operations in their judgment and decision-making. There were also differences in thinking style, with traders indicating a higher tendency to reflective/analytic thinking compared to non-banking participants. However, no such difference was found for experientiality (or ‘intuition’): Traders in the current sample showed no more (or less) inclination to rely on their ‘intuitions’ or gut feelings in their decision-making compared to other groups, so disproving our second hypothesis. This was despite the fact that experientiality correlated positively with age across the sample and with years of traders’ experience in Study 1, indicating that the REI-E indeed measures attitudes implicated in intuitive thought (e.g., [51]) theories.

These results are important as Study 1 found that cognitive style also correlated with financial risk-taking, a variable that is central to the work as a financial expert. Indeed, we found that both traders and non-trading bankers had elevated financial risk-taking scores compared to the non-expert sample. The observed differences between participant groups cannot be attributed to factors such as age, sex, or general level of education. The finding that at least a part of traders’ expertise relies on the ability to engage in reflective/analytic thinking, is not trivial, given results from previous studies in other domains (e.g., [52], [53]) in which experts seem to rely on fast, automatic processing. Our findings also challenge previous reports that successful financial experts are using intuitive decision-making strategies successfully [35], [36]. The lack of difference in experientiality between groups seems also interesting given that biases based on ‘experiential’ phenomena (most commonly heuristics and ‘overconfidence’) in financial decision-making have been frequently reported in economic [54]–[56] as well as behavioural studies [57], [58].

Of course, our data do not necessarily mean that financial decision-makers are not using ‘intuition’ in certain situations or tasks. Indeed, we did not measure directly whether actual trading-related decision problems are solved heuristically or analytically. Crucially, however, we found no evidence (from CRT or REI scores) that traders primarily utilised System 1 (experiential) processes or relied more on ‘intuition’ than non-traders. In fact, the CRT results support the opposite conclusion. However, higher CRT scores are generally connected to other individual differences as well, notably cognitive ability. The CRT has been shown to correlate with measures of general cognitive ability [23], and it is possible that the trader sample reflects the now rigorous screening of job applicants which selects for high-performing graduates from prestigious universities, explaining the negative correlation between REI-R scores and years of experience [37].

To address the point of whether group differences in cognitive ability alone can explain the current results, it is important to note that correlations between the CRT and measures of cognitive ability have been found to be at best moderate [23], [24]. There are also indications in the current data that speak against the interpretation that the differences in CRT scores for traders are solely a consequence of having higher cognitive ability. First, the samples did not differ in regards to levels of qualification, and there was no correlation between levels of education and CRT. Inasmuch as the level of education can be considered a proxy for cognitive ability [59] this implies that the differences in CRT scores between traders and the other groups were not due to a simple difference in cognitive ability. Second, after analysing only high-scoring (and therefore presumably cognitively high-performing) male participants who had at least 2 out of 3 CRT questions correct, traders still outperformed the other groups. Third, across the whole sample, CRT scores co-varied significantly with the tendency to follow a reflective approach (REI-R) to decision-making. Although a contributing effect of higher cognitive ability on CRT scores cannot be ruled out, together these patterns in the data lend weight to the conclusion that at least a substantial part of the variance in CRT scores is related to the tendency of engaging a reflective/analytic approach and monitoring (and possibly inhibiting) heuristic (i.e. ‘intuitive’) responses that is independent of cognitive ability.

These conclusions also fit with the observations of Stanovich and West [18] who have shown that intelligence is not, or only weakly, related to performance on tasks exhibiting heuristic thinking. Recently, Toplak and colleagues [24] showed that the CRT predicted use of heuristic thinking even when measures of cognitive ability and executive functioning were controlled for. They suggest that tests of intelligence or cognitive aptitude are optimal performance assessments, whereas measures of thinking styles are usually assessed under typical performance conditions [60] and the CRT typically correlates with both. Yet the relationship between typical and maximal performance is rather weak across a variety of domains (see [24] for a discussion) and research in other areas of expertise such as in music (e.g., [61]) and chess (e.g., [62]) has found that experts and non-experts perform differently despite scoring equivalently on intelligence scales. Toplak et al. therefore conclude that the CRT measures rational ability beyond general intelligence. This view is similar to conclusions by Campotelli and Labollita [63] who investigated the impact of individual differences on cognitive reflection. They proposed that cognitive reflection is a thinking disposition and supported Frederick’s [23] view that correlation in decision-making tasks and CRTs cannot be explained simply by individual differences in general knowledge and cognitive abilities alone. Rather, cognitive reflection is specifically related to the ability to override System 1 processes and selectively initiate System 2 processing in relevant situations.

Related to the issue of cognitive ability, it could be argued that the CRT is just another measure of numeracy. This would potentially also account for known correlations of the CRT with increased selections of risky (gain) options. Frederick [23] found with large samples a rather small (*r* ~ 0.2) correlation between CRT and selecting gain options in gambles, and indeed across all participants in the current investigation we found a significant correlation (r = 0.2) between CRT and risk-taking scores (there was no such correlation in the trader group but this could be due to the high number of top scores). However, there is evidence that the CRT measures constructs other than numeracy, which in itself comprises several distinctive factors [42].

There is a further observation in our data indicating that traders’ superior CRT scores are not because of cognitive ability alone. Responses to the REI scales indicate that a preference for ‘intuitive’ thinking (REI-E) correlated negatively with ‘rationality’ (REI-R) in the trader group—a result which apparently contradicts previous findings that scores on these two scales (and by extension the two styles) are independent of each other (Epstein & Pacini, 1996). Indeed, across the whole sample there was no significant correlation of these subscales of participants. One interpretation of this pattern of results is that traders suppress experiential thinking to a substantial degree and find it advantageous to favour an analytical style. This conclusion seems supported by findings [64] that scores on the REI rationality scale correlate negatively with choice framing effects [65], further supporting the evidence for the validity of the REI-R (see also [29]) as a measure for rational thinking. Indeed, we observed that in the non-trader groups rationality correlated with CRT scores (there was no potential issue of a ceiling effect here), validating the REI-R as a subscale related to System 2 processes.

Our interpretation of the pattern of results is therefore that one reason for the current data is that our trader sample is scoring higher on the CRT because of a more reflective/analytical approach to judgment and decision-making and not just simply because of their higher cognitive ability alone. What is the reason for this? Toplak et al. [24] discuss the argument that people in general are cognitive misers [2], [6] meaning that human beings usually follow strategies that make the task at hand cognitively easier, an argument that is at the heart of the heuristics and biases literature [4]. According to Toplak et al., however, being a cognitive miser in modern technological societies may impede achieving a goal. As such, traders may have learned to expend more effort and engage in reflective thinking because their work requires it. Campotelli and Labollita propose a similar idea when interpreting their work on the relation between CRT scores and decision-making tasks as showing that cognitive reflection is linked to “open-minded thinking”—meaning a disposition for searching for more possibilities before making inferences [66]. However, there was no significant correlation between CRT scores and length of work experience, and even a negative correlation with REI-R and experience (although this may reflect other factors, such as a cohort effects of job selection [37]). Furthermore, the difference in REI-R scores between traders and bankers was not significant. Thus, further research may further elucidate the reason for traders’ higher propensity in reflective style (e.g, individual differences, recruitment selection, survival, or adaptation).

The current data were interpreted in the dual systems framework suggesting that judgment and decision-making can be conceptualised as stemming from two qualitatively different Systems (or processes), usually labelled System 1 and System 2 (e.g., [3], [17]). There is a consensus that this distinction provides a valuable framework in which to investigate models of human decision-making [3], [18], [67]. Dual-process theories have been used to explain other psychological constructs, such as object recognition [68], social cognition [69], and memory processes [70]. However, there is a debate about whether at least some of the observed phenomena in the decision-making literature are not better conceptualised as dimensions of a hybrid or unitary system [71] or a collection of specialised modules [38]. It is worth noting that the CRT specifically has been developed in the tradition of Kahneman and Tversky’s heuristics and biases experiments in which performance was measured against the standards of logic and probability theory. This approach has been criticised by researchers who claim that the biases demonstrated by asking participants somewhat artificial questions disappear when tested in real-life environments [5]. Similarly, theorists in the areas of intelligence and problem-solving have debated the role of domain-specific vs. domain independent processing of task expertise [72]. The current results showing superior CRT and REI-R scores for traders and no difference in experientiality between groups are arguably more difficult to explain by theories that propose expert intuition as purely rooted in domain-specific knowledge [52].

What are the practical implications of the present data? Biais and Weber [73] found that financial traders in London and Frankfurt were more successful (i.e., profitable) when they were shown to exhibit fewer biases (such as the hindsight bias). In their study, the heuristics were investigated with finance-related questions, but it is tempting to speculate—given Toplak et al.’s [24] and the present findings—that scales such as the CRT and REI could be utilised to develop more sophisticated predictors for financial experts’ success. Note that while the present results seem to imply a positive message in terms of traders’ superior ability in a profession whose actions affect an important part of the economy and in consequence, the society at large, the generalisation of these results is as of yet limited due to the relatively small size of our particular sample (and the inherent limitations of finding a trader sample that can be considered representative for the profession as a whole). Furthermore, although the majority of our traders did very well on the CRT, still 12% scored poorly (correctly answering only one or none of the questions). If these results are replicated with larger samples (also controlling for other potential confounding variables related to cognitive functioning; e.g. medication), future research could investigate if situational factors (e.g., their specific role, say, as a market maker) impact on traders and their tendency to follow type 2 thinking, and why and when financial experts are motivated or pre-disposed to override heuristic responses. Importantly, a next step would be to determine which real-life performance indicators for traders and similar professions correlate with the CRT and other rationality measures.

In conclusion, the current study concurs with recent work [24], [63] that the CRT appears to be a valid measure for rational thinking, as across all participants it correlates with rational thinking style (REI-R). While traders are apparently no different in their use of ‘intuition’ or ‘gut feeling’ to other people, the majority of them are markedly improved in their propensity to engage in reflective thinking and less susceptible to ‘cognitive impulsivity’ [23]. Future studies will have to establish to which degree this tendency is due to ability or cognitive style, and how it is related to performance in the market place.

## Supporting Information

S1 Data Explanation of data files. (DOCX)

S2 Data Data file for Study 1. (CSV)

S3 Data Data file for Study 2 (CSV)
