# Supplementary material for: Good Thinking or Gut Feeling? Cognitive Reflection and Intuition in Traders, Bankers and Financial Non-Experts
Source: PLoS One. 2015 Apr 13;10(4):e0123202. doi: 10.1371/journal.pone.0123202 (PMC4395391; doi:10.1371/journal.pone.0123202)
Supplement: S1 Data — (DOCX) [file pone.0123202.s001.docx]

Explanation of data files Thoma et al.

1. File 1 “Study1_44traders” contains the data for the Study 1 with 44 traders.
2. File 2 (“Study2_all_grous”) contains data from all groups comprising all participants. Results for Study 2 were used by filtering for Groups.

NOTE: Values of “999” are missing data

Explanation of Variables:

Qualific: Level of education 1 = lowest (no GSCE)

Activity: Profession (e.g. trader)

CRT: Score for Cognitive Reflection test

NFC_or_Rational: REI score for scale “Rationality” (Need for Cognition)

FI_or_Experiential: REI score for scale “Experientiality” (Faith in Intuition)

FinRisk: Score for Financial risk-taking (1 = low)

nonFinRisk Score for no-financial risk-taking (1 = low)

Salaryandbonus: Salary including Bonus (trader only)

AvTxperday: number of average transactions per day

Years_experience: number of years’ experience as professional trader

Study 2:

An extra 2 columns are used for the 2 between group analyses (one in Study 2 only, and one across all 3 groups of participants). For study 2, filter “Groups_S2” for ‘2’ (banking) and ‘3’ (non banking).
